# Continuous integrated antibody precipitation with two‐stage tangential flow microfiltration enables constant mass flow

**DOI:** 10.1002/bit.26922

**Published:** 2019-01-23

**Authors:** Daniel Burgstaller, Alois Jungbauer, Peter Satzer

**Affiliations:** ^1^ Department of Biotechnology University of Natural Resources and Life Sciences, Vienna Austria; ^2^ Austrian Centre of Industrial Biotechnology (ACIB) Vienna Austria

**Keywords:** diafiltration, IgG, membrane, polyethylene glycol, tubular reactor

## Abstract

Continuous precipitation is a new unit operation for the continuous capture of antibodies. The capture step is based on continuous precipitation with PEG6000 and Zn^++^ in a tubular reactor integrated with a two‐stage continuous tangential flow filtration unit. The precipitate cannot be separated with centrifugation, because a highly compressed sediment results in poor resolubilization. We developed a new two‐stage tangential flow microfiltration method, where part of the concentrated retentate of the first stage was directly fed to the second stage, together with the wash buffer. Thus, the precipitate was concentrated and washed in a continuous process. We obtained 97% antibody purity, a 95% process yield during continuous operation, and a fivefold reduction in pre‐existing high‐molecular‐weight impurities. For other unit operations, surge tanks are often required, due to interruptions in the product mass flow out of the unit operation (e.g., the bind/elute mode in periodic counter‐current chromatography). Our setup required no surge tanks; thus, it provided a truly continuous antibody capture operation with uninterrupted product mass flow. Continuous virus inactivation and other flow‐through unit operations can be readily integrated downstream of the capture step to create truly continuous, integrated, downstream antibody processing without the need for hold tanks.

## 1 INTRODUCTION

Continuous and batch precipitation methods were described by our group and others as an economically viable option for the continuous capture of antibodies from clarified culture supernatants (Grosshans, Wang, Fischer, & Hubbuch, 2018; Hammerschmidt, Hintersteiner, Lingg, & Jungbauer, 2015; Hammerschmidt, Hobiger, & Jungbauer, 2016; Hammerschmidt, Tscheliessnig, Sommer, Helk, & Jungbauer, 2014; Kateja, Agarwal, Saraswat, Bhat, & Rathore, 2016; Rathore, Kateja, Agarwal, & Sharma, 2016; Tscheliessnig et al., 2014). This method is quite robust; it readily accommodates varying feed concentrations, and it can be combined with flocculation. Converting the process into a continuous operation is relatively simple with tubular reactors (Burgstaller et al., 2018; Hammerschmidt et al., 2016; Hekmat, Huber, Lohse, von den Eichen, & Weuster‐Botz, 2017; Kateja et al., 2016; Raphael & Rohani, 1999). However, one problem that remains unsolved is the continuous solid–liquid separation step, which comes after the actual precipitation, particularly when the solid fraction is the desired product. Centrifugation is an option for continuous solid–liquid separation, but it is difficult to realize for the continuous harvest of polyethylene glycol (PEG)‐precipitates. We have observed that extreme compaction of the precipitate is detrimental to the process yield, because the dissolution of dense precipitates requires vigorous stirring and shaking, and the air–liquid interphases generated cause protein aggregation and denaturation. In contrast, the precipitation reaction is a gentle technology that provides high yield; therefore, we must develop new, gentle continuous solid–liquid separation and dissolution methods to establish a continuous antibody precipitation process with high yield.

One way to avoid compaction of the precipitate is membrane filtration, which avoids harsh resolubilization conditions altogether. Continuous membrane filtration can be realized by running at least two staggered filtration processes. However, that strategy interrupts the fully continuous operation, and the antibody must be harvested in cycles. Another possibility is single‐pass tangential flow filtration (TFF), which allows the solid to be harvested in a single pass through a cascade of membrane filters (Casey, Gallos, Alekseev, Ayturk, & Pearl, 2011; Rucker‐Pezzini et al., 2018). The third choice is counter‐current TFF. This method is used for extremely large‐scale production in other industry sectors, like oil processing. Counter‐current TFF can also be readily connected to continuous diafiltration, which can be used for washing and/or dissolution of the precipitate (Jungbauer, 2013; Nambiar, Li, & Zydney, 2018).

In the present study, we focused on antibody precipitation with PEG as the capture step. Previous studies have shown that PEG with a molecular mass of 6,000, at a concentration in the range of 14%, provided a good tradeoff between yield and viscosity in process solutions (Grosshans et al., 2018; Hammerschmidt et al., 2015; Hammerschmidt et al., 2016; Sommer et al., 2014, 2015). Although the amount of PEG can be reduced by increasing the molecular mass, this causes an increase in the viscosity. High viscosity can be a problem in certain unit operations, particularly when membrane filtration is used. The upper limit of the transmembrane pressure governs the maximum achievable flux. Accordingly, the cross‐flow velocity of highly viscous fluids is low, and consequently, the mass transfer rate through the membrane is slow. These engineering relationships for optimizing membrane filtration have been well established and dictate that lower viscosity is beneficial for increasing the flow rate. Therefore, reducing the viscosity is a critical goal for an efficient process.

It has been shown that the addition of a bivalent cation, such as zinc, might substantially reduce the PEG concentration needed for complete precipitation of the product (Przybycien, [Link]). Indeed, Zn^++^ bridges the protein molecules in a flocculent‐like manner, therefore, it can reduce the necessary PEG concentration (Iyer & Przybycien, 1995; Yang et al., 2000). We observed that even at neutral pH, where the net charge of our antibody is positive, this method works, presumably because of bridging effects in between negative batches of the antibody. A combination of Zn^++^ and PEG could greatly reduce the viscosity of the PEG solution, which would be very beneficial for membrane filtration. PEG precipitation can be used as a platform process, but to obtain high yields, the exact precipitation and resolubilization conditions must be fine‐tuned for each antibody to optimize the process for maximum productivity. Currently, this optimization can be accomplished with high throughput screening methods in microtiter plates (Grosshans et al., 2018).

Precipitation is directly scalable to any size. The necessary equipment is readily available for all manufacturing scales, and the method is highly stable and largely independent of the concentration of product in the feed stream. When the protein concentration is within a broadly constant range, there is no need to adjust the precipitant concentration, and the unit can be run without including on‐line measurements of product concentrations, which simplifies the method and avoids expensive equipment and measurements. In the presence of host cell proteins (HCP), product concentration determinations are very tricky; even the best current technologies are limited in this application (Brestrich, Rüdt, Büchler, & Hubbuch, 2018; Jiang et al., 2017). In the present study, we optimized precipitation and dissolution conditions to mitigate problems caused by high viscosity due to PEG concentrations. We also developed a novel method for connecting and running multiple TFF unit operations to facilitate continuous harvesting, concentrating, and washing procedures for a given precipitate. This method can be directly integrated into any production facility and continuous process sequence.

## 2 EXPERIMENTAL

### 2.1 Cell culture

Immunoglobulin G1 was produced in Chinese hamster ovary (CHO) cells in a 1000 L fed‐batch fermentation. For the primary separation, one fraction of the fed‐batch (cell density 7.5*10^6^ cells/ml, cell viability 91.7%) was filtered (Millistak+^®^ D0HC/F0HC+ Millipore Express^®^ SHC in‐line; Merck KGaA, Darmstadt, Germany) on Day 1. The second fraction of the fed‐batch (cell density 7.3*10^6^ cells/ml, cell viability 89.3%) was flocculated with 0.0375% of the polycationic polymer, polydiallyldimethylammonium chloride (pDADMAC; Merck KGaA) and filtered (Clarisolve^®^ 40MS+ Millipore Express SHC in‐line; Merck KGaA) on Day 2. The cell culture broths were kindly provided by LEK, a Sandoz company (Ljubljana, Slovenia)

### 2.2 Precipitant optimization

For buffer and pH optimization, 50 mM phosphate buffer (Merck KGaA) in a pH range of pH 6.0 to 8.0 and 50 mM tris(hydroxymethyl)aminomethane buffer (Merck KGaA) in a pH range of pH 7.0 to 9.0 were tested in the presence of 8 to 12% PEG (PEG6000, average molecular weight, 6000 g mol^−1^; Merck KGaA). Before testing, the cell culture broth (with the desired antibodies) was adjusted to the appropriate pH value with either hydrochloric acid (25% HCl) or sodium hydroxide (10 M NaOH). In a second round, we optimized the addition of zinc chloride (ZnCl_2_), in the concentration range of 1 to 10 mM, and sodium chloride, in the concentration range of 50 mM to 150 mM, in 50 mM Tris buffer with pH 7.0. No pH adjustment of the cell culture broth was required. Finally, solubility curves were created over a range of 0 to 15% PEG6000, with and without the addition of 2 mM ZnCl_2_. All optimization experiments were performed in 96 deep‐well plates. After 20 min of incubation at room temperature on the end‐over‐end shaker (Stuart rotator SB3; Cole‐Parmer, Vernon Hills, IL), the plate was centrifuged at 4,000rcf for 10 min (Centrifuge Heraeus Multifuge X3, Rotor HIGHPlateTM6000; Thermo Fisher Scientific, Waltham, MA). The supernatant was withdrawn and analyzed with protein A affinity chromatography (described in section 2.9). All PEG6000 concentrations are defined in terms of weight per volume (w/v) unless stated otherwise.

### 2.3 Resolubilization optimization

The antibody was precipitated for 20 min in a 100‐ml stirred vessel by adding 15 ml of 40% PEG6000 in 50 mM Tris buffer, pH 7.0, 34.8 ml 50 mM Tris buffer, pH 7.0 and 200 µl 1 M ZnCl_2_ solution to 50 ml of cell culture broth to gain a final concentration of 6% PEG6000 and 2 mM ZnCl_2_ in 50 mM Tris buffer, pH 7.0. The precipitate was washed twice. Next, the precipitate was centrifuged at 2,000rcf for 5 min. The supernatant was withdrawn, and an equal volume of 6% PEG6000 solution with 2 mM ZnCl_2_ in 50 mM Tris buffer, pH 7.0 was added. The pellet was resuspended and adjusted to the particular pH (pH 3.5–pH 8.0) by titration with 25% HCl. For the buffer and pH optimizations, the pH‐adjusted, resuspended precipitate was added, according to its pH value, to either 50 mM sodium acid buffer (pH 3.5–pH 6.0) or 50 mM phosphate buffer (pH 6.0–pH 8.0) at dilution ratios of 1:2, 1:3, and 1:5. In the second step, we evaluated samples to determine the influence of adding 0 to 150 mM ammonium phosphate and sodium chloride. All experiments were performed in 96 deep‐well plates. After incubating for 20 min at room temperature on the end‐over‐end shaker, the samples were analyzed with protein A affinity chromatography (described in section 2.9).

### 2.4 Sequential TFF–proof of concept

The cell culture broth was combined with 40% (w/w) PEG6000 in 100 mM Tris buffer, pH 7.5 to achieve a final 13.2% (w/w) PEG6000 (Grosshans et al., 2018). The precipitate suspension was incubated for 20 min in a stirred vessel. Next, it was continuously pumped via a transfer pump at a flow rate of 15 ml min^−1^ into the stirred tank vessel of an Äkta flux S system (GE Healthcare, Uppsala, Sweden). In the first stage, the precipitate was concentrated by passing it through a 50 cm² hollow fiber membrane (pore size, 0.2 µm; GE Healthcare) at a constant feed rate of 124 ml min^−1^ and a constant permeate flow of 13.8 ml min^−1^. A constant bleed was withdrawn at a flow rate of 1.2 ml min^−1^. After 185 min of run‐time, the transfer of the precipitate suspension was terminated. The precipitate was further concentrated for 35 min until a feed pressure of 170,000 Pa was achieved. In the second stage, the withdrawn precipitate was combined with the precipitate that remained in the Äkat flux S system (310 ml) and washed with 13.2% (w/w) PEG6000 in 100 mM Tris buffer, pH 7.5 (2690 ml). In this washing stage, we applied the same conditions as those used in the first stage. The precipitate feed was terminated after 190 min of run‐time and further concentrated for 10 min until a feed pressure of 170,000 Pa was achieved. Samples were prepared and diluted for size exclusion chromatography analysis.

### 2.5 Influence of residence time in continuous precipitation with tubular reactors

Continuous precipitation was achieved with a self‐assembled tubular reactor setup (described in section 2.7). The cell culture broth was combined with 40% (w/w) PEG6000 in 100 mM Tris buffer, pH 7.5, with a peristaltic pump (Ismatec ISM597D; Cole‐Parmer) to achieve a final precipitation condition of 13.2% (w/w) PEG6000 (Grosshans et al., 2018). The hold time (residence time) in the tubular reactor was varied within a range of 2.5 to 22.5 min, by adapting the length of the tubular setup. The run‐time of the continuous precipitation was varied up to 160 min. Samples were taken at various time points and analyzed with size exclusion chromatography. We calculated the standard deviations of yields, antibody purities, and high molecular weight impurities (HMWI), based on experimental repetitions and sampling at various time points.

### 2.6 Influence of membrane pore size

The cell culture broth was combined with 40% PEG6000 plus 11.43 mM ZnCl_2_ in 50 mM Tris buffer, pH 7.0 to achieve a final precipitation condition of 7% PEG6000 with 2 mM ZnCl_2_ in a volume of 500 ml. The stirred tank of the Äkta flux S system (GE Healthcare) was used as a reactor vessel. The stirrer speed was set to 250 rpm. After 30 min of hold time, the precipitate was concentrated on a 50‐cm² hollow fiber module fit with a filter membrane (either 0.1 or 0.45 µm pore size; GE Healthcare), until a feed pressure of around 100,000 Pa was achieved. The feed flow rate was set to 200 ml min^−1^, and the permeate flow rate was set to 7.2 ml min^−1^.

### 2.7 Continuous precipitation

Continuous precipitation was achieved with a self‐assembled tubular reactor setup. Spirally‐arranged standard lab tubes (Tygon^®^ R‐3603, 4.8‐mm inner diameter; Saint‐Gobain, Courbevoie, France) filled with static mixers (HT‐40‐6.30‐24‐AC; Material Acetal; Stamixco AG, Wollerau, Switzerland) were vertically stacked. The setup was connected with polycarbonate Luer fittings (Cole‐Parmer). The cell culture broth was continuously pumped at a flow rate of 6.6 ml min^−1^ (transfer pump stage one) and combined with a feed stream of 1.4 ml min^−1^ (permeate pump stage one) of 40% PEG6000 plus 11.43 mM ZnCl_2_ in 50 mM Tris buffer, pH 7.0. This ratio resulted in precipitation conditions of 7% PEG6000 with 2 mM ZnCl_2_. The hold time in the tubular reactor was 22.5 min at this flow rate. For continuous operation, two Äkta flux S (GE Healthcare) setups were connected in series. The precipitate was continuously fed into stage one and concentrated in the 0.2‐µm hollow fiber module (GE Healthcare) with a filter area of 420 cm². In stage two, the precipitate was washed with the continuous addition of 7% PEG6000 with 2 mM ZnCl_2_ in 50 mM Tris buffer, pH 7.0 and concentrated in the 0.2‐µm hollow fiber module (GE Healthcare) with a filter area of 110 cm². Both hollow fiber modules were run at a 400 ml min^−1^ feed rate. The permeate flow rate was 7.2 ml min^−1^. An external peristaltic pump (Ismatec ISM 597; Cole‐Parmer) with a flow rate of 0.8 ml min^−1^ was used to pump the concentrated precipitate continuously, from stage one to stage two, and to withdraw the washed precipitate continuously out of stage two. A second external peristaltic pump (Ismatec IPC; Cole‐Parmer), with a flow rate of 7.2 ml min^−1^, was used at the permeate site in stage one. For preliminary experiments, we used hollow fiber modules of various dimensions (e.g., 50 cm² with 0.1‐µm, 0.2‐µm, and 0.45‐µm filters; GE Healthcare).

### 2.8 Sample preparation for analytics

The precipitate was centrifuged at 2,000rcf for 1 min (Eppendorf centrifuge 5415R; Eppendorf, Hamburg, Germany). Then, the supernatant was withdrawn, and an equal volume of resolubilization buffer was added (gravimetrically determined). After resuspension and dilution, the samples were filtered through 0.2‐µm filters before injection onto a high‐performance liquid chromatography (HPLC) column.

### 2.9 Protein A affinity chromatography

Protein A affinity chromatography was used to determine the antibody concentration. For HPLC, we used a Dionex UltiMate 3000 HPLC system equipped with a diode array detector (Thermo Fisher Scientific). Mobile phase A was a 50 mM phosphate buffer with 150 mM NaCl, pH 7.0. Mobile phase B was a 100 mM glycine buffer, pH 2.5. All buffers were filtered through 0.22‐µm filters (GSWP04700; Merck KGaA) and degassed. The system was run at a flow rate of 2.5 ml min^−1^. We loaded 20 µl of the sample, filtered through 0.2‐µm filters (0.2‐µm GHP AcroPrepTM 96 filter plate; Pall Life Sciences, Ann Arbor, MI), on a POROS A 20 µm Column (2.1 × 30 mm, 0.1 ml; Thermo Scientific). The column was washed with 10 column volumes of mobile phase A, eluted with 20 column volumes of 100% mobile phase B, and re‐equilibrated with 30 column volumes of mobile phase A. The absorbance at 280 nm was measured. We used a similar protein A‐purified IgG1 as the calibration standard. The calibration range was 0.1 to 3 mg mL^−1^. We evaluated and quantified the results with the Chromeleon^TM^ 7 software (Thermo Fisher Scientific).

### 2.10 Size exclusion chromatography

We used size exclusion chromatography to determine the soluble HMWI and to estimate the product purity. For HPCL analytics, we used a Dionex UltiMate 3000 HPLC system equipped with a diode array detector (Thermo Fisher Scientific). The running buffer was a 50 mM sodium phosphate buffer with 150 mM NaCl, pH 7.0 (Merck KGaA). The buffer was filtered through 0.22‐µm filters (GSWP04700; Merck KGaA) and degassed. After filtering the sample through 0.2‐µm filters (0.2‐µm GHP AcroPrepTM 96 filter plate; Pall Life Sciences), we applied 10 µl of the sample to a TSKgel^®^ G3000SWXL HPLC Column (5 µm, 7.8 × 300 mm) in combination with a TSKgel SWXL Guard Column (7 µm, 6.0 × 40 mm; Tosoh, Tokyo, Japan). The absorbances at 215 nm (for HMWI determinations) and 280 nm (for purity estimations) were recorded, and the results were evaluated with the Chromeleon^TM^ 7 software (Thermo Fisher Scientific). The antibody purity was calculated as the ratio of the monomer peak area to the sum of all peak areas, based on the 280 nm signal. The HMWI content was calculated as the ratio of the HMWI peak area to the sum of the monomer peak area plus the HMWI peak area, based on the 215 nm signal. This method just determines the soluble HMWI. Insoluble aggregates will be filtered out and not be detected. When ZnCl_2_ was present in the sample, a wash step was performed to remove ZnCl_2_, because ZnCl_2_ seemed to interact with the size exclusion chromatography column media. Briefly, 350 µl of sample was pipetted into Amicon^®^ spin tubes with a molecular weight cut‐off of 50 kDa (Merck KGaA). The spin tubes were placed in 1.5 ml reaction tubes and centrifuged at 16,100rcf for 5 min (Eppendorf centrifuge 5415R; Eppendorf). Then, 350 µl of 50 mM sodium acid buffer, pH 3.5 (resolubilization buffer), was added to the sample, and the sample was centrifuged again. This buffer exchange procedure was repeated five times.

## 3 RESULTS AND DISCUSSION

### 3.1 Optimization of precipitation conditions

**Figure 1:**
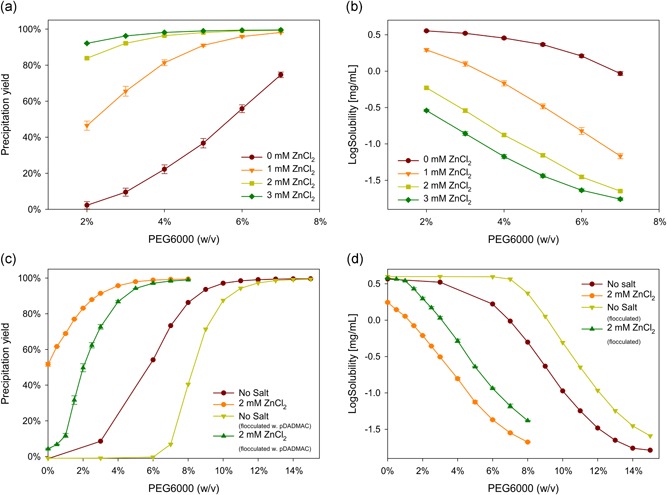
Optimization of antibody precipitation. (a) Precipitation yield with increasing PEG and ZnCl_2_ concentrations; (b) solubility curve for increasing PEG and zinc concentrations; (c) influence of flocculation and added salt on precipitation yield; (d) resulting apparent solubility. The experiments were performed at pH 7.0. PEG: polyethylene glycol [Color figure can be viewed at wileyonlinelibrary.com]

To optimize the precipitation protocol for this specific antibody, we fine‐tuned the precipitation conditions in microtiter plates. In a previous study, we found that the optimal PEG size for antibody precipitation was PEG6000 (Sommer et al., 2014). Therefore, here, we tested different PEG6000 concentrations. We also tested the effects of adding ZnCl_2_ to the feed material and the effects of flocculation before precipitation. Flocculation was described in a previous study (Burgstaller et al., 2018). In short, pDADMAC was used for cell flocculation, and cells were removed with depth filtration. In an initial test, we determined the optimal concentration of ZnCl_2_ to add to the clarified culture supernatant (Figure 1a). We found that 7% PEG combined with 2 mM ZnCl_2_ precipitated more than 99% of the antibody. However, higher ZnCl_2_ concentrations seemed to give diminishing returns. In a second test, we assessed the influence of prior flocculation on antibody precipitation; for precipitation, we used 2 mM ZnCl_2_ and different PEG6000 concentrations. Interestingly, preflocculated material required a higher PEG concentration than unflocculated material to precipitate the same amount of antibody. When pDADMAC was used in flocculation, host DNA coflocculated with the cell debris (Burgstaller et al., 2018; Tomic et al., 2015). We previously showed that an antibody and DNA coprecipitated during PEG precipitation (Hammerschmidt et al., 2016; Sommer et al., 2015). Accordingly, we speculated that the coprecipitation of DNA with an antibody could explain why a lower PEG concentration was required for unflocculated material. In theory, the solubility of the antibody should be independent of the feed solution; however, due to this coprecipitation, we will subsequently refer to the solubility as an “apparent solubility.” For both flocculated and unflocculated materials, the addition of ZnCl_2_ greatly reduced the apparent solubility; 7% PEG was sufficient for precipitating unflocculated material, and 12% PEG was required for precipitating flocculated material. Viscosities of 7 to 12% PEG6000 solutions are amenable to pharmaceutical downstream processes. The addition of ZnCl_2_ is a very common practice in different unit operations for bioprocessing. Similar zinc concentrations are found in other settings. For example, in‐house CHO cell media contains 0.7 to 1.6 mM Zn^++^, and standard cell culture media, such as Dulbecco's modified Eagle's medium /Ham's F‐12 CHO media, contains 0.432 mM Zn^++^ (Wong, Ho, & Yap, 2004).

**Figure 2:**
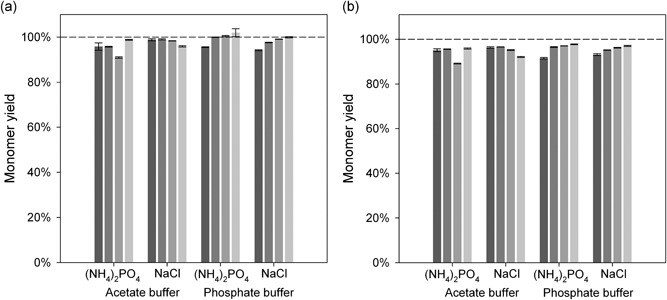
Effects of added salts on antibody monomer yields in optimized antibody precipitation and resolubilization conditions. Resolubilization was performed with 50 mM acetate buffer, pH 3.5, at a dilution of 1:2 or with 50 mM phosphate buffer, pH 7.0, at a dilution of 1:5, with or without added (NH_4_)_2_PO_4_ or NaCl. Increasing salt concentrations are indicated with decreasing gray shading (dark gray: 0 mM to light gray: 150 mM; salt added) (a) Antibody monomer yield from nonflocculated cell culture broth under various resolubilization conditions. (b) Antibody monomer yield from flocculated cell culture broth under various resolubilization conditions

The key to a successful precipitation process is an efficient resolubilization buffer. A common method for resolubilization is to dilute the precipitate in various buffers. However, to maintain low process volumes, we tested new resolubilization buffers to optimize the dilution ratio. In an initial test, we evaluated a 50 mM sodium acetate buffer at various pH levels (3.5, 5.0, and 6.0) and a 50 mM phosphate buffer at pH 6.0, pH 7.0, and pH 8.0. These buffers were tested at different dilution ratios of 1:2, 1:3, and 1:5. Then, the best conditions for each buffer system were used for the second round of optimization. For the acetate buffer, the best condition was pH 3.5 at a dilution ratio of 1:2; it provided a 91% resolubilization yield. For the phosphate buffer, the best condition was pH 7.0, at a dilution ratio of 1:5; it provided a 94% resolubilization yield. For both buffer systems, we attempted further optimization of the resolubilization yield by adding ammonium phosphate and sodium chloride in different concentrations (Figure 2). Based on these results, we determined that optimal resolubilization was achieved with 50 mM sodium acetate buffer at pH 3.5 without the addition of salt. The phosphate buffer achieved a slightly higher resolubilization yield than the acetate buffer, but the phosphate system was based on a 1:5 dilution, compared with the 1:2 dilution required for the acetate system. Because our objective was to concentrate the antibody solution as much as possible, we selected the 1:2 dilution with the acetate system. In addition, the acetate buffer required as little salt addition as possible. This feature provided an advantage for subsequent polishing steps, such as ion exchange chromatography. Moreover, a pH of 3.5 would be advantageous for a subsequent viral inactivation at low pH, which is commonly performed after the capture step in antibody purification. Thus, the low pH, minimal dilution, and minimal salt addition conditions that provided efficient resolubilization were expected to fit well in any commonly used antibody purification scheme, without further modification.

### 3.2 Sequential TFF

We used TFF for harvesting, concentrating, and washing the precipitate, because TFF does not compress the precipitate, compared with centrifugation or dead‐end filtration. Compressed precipitates have very slow resolubilization kinetics because the resolubilization buffer cannot penetrate deeply into the compressed structure of the precipitate. Avoiding compaction of the precipitate facilitates resolubilization significantly and reduces the process time. A viable, integrated capture step for continuous operations requires volume reduction, contaminant removal, and a mode of operation transfer from batch TFF.

**Figure 3:**
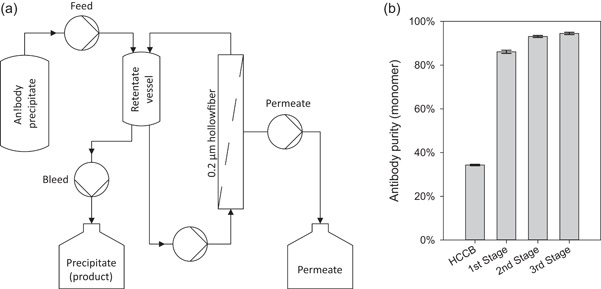
Sequential tangential flow filtration (TFF)/discontinuous diafiltration. (a) Flow diagram of the sequential TFF. (b) The purity of the antibody monomer: before TFF, in the host cell culture broth (HCCB); after the antibody precipitate concentration (1st Stage); after the first antibody precipitate wash step with continuous diafiltration (2nd Stage); and after the second antibody precipitate wash step (3rd Stage)

Initially, we tested the feasibility of the intended continuous integrated TFF method in sequential operations (Figure 3a). In each stage, the precipitate was fed with a constant flow rate from a tank into the retentate vessel of the TFF unit. A controlled bleed flow from the retentate vessel was collected, and the material that permeated the membrane went to waste. The feed and bleed flow rates remained constant. The permeate flow rate was adjusted to maintain a constant volume in the retentate vessel. The bleed stream contained the concentrated antibody product. This product was then diluted with wash buffer and used to feed the next stage. With this approach, we conducted several sequential stages of concentrating and washing the precipitate with a single bench‐top instrument.

For the three sequential stages (one concentration stage and two wash stages), we used the same 0.2‐µm hollow fiber membrane with a tangential flow of 675 L m^−1^h^−1^ (LMH; Hammerschmidt et al., 2016) for concentration. The permeate flow was regulated to 75 LMH (Hammerschmidt et al., 2016) with a pump. At the retentate vessel, the ratio of bleed flow:feed flow was 1:12.5, which resulted in a theoretical concentration of 12.5‐fold, once the unit reached a steady state. After each stage, the collected, concentrated antibody precipitate slurry was diluted (1:10) with wash buffer, and subsequently, it was concentrated again in the next unit. This procedure mimicked a continuous process with a step‐wise diafiltration process to remove nonprecipitated contaminants. After each such stage, the precipitate was dissolved and measured with size exclusion chromatography for purity (Figure 3b). We observed that, after the second stage, the purity did not increase further; therefore, we selected a two‐stage process for the continuous operation. Moreover, the size exclusion chromatography results showed that no additional aggregation occurred, compared to that observed in the cell culture broth.

During the run‐time, we also determined the concentration of antibody in the retentate vessel. We found that the concentration varied over the run‐time of 200 min, and it did not reach a steady state within this period. To estimate the time to steady state for the concentration and wash steps, we setup a simple mass balance model. The feed concentration of our precipitated antibody and all flow rates of the system were known. We summed the accumulated antibody in the permeate vessel and in the bleed stream at each time point of the process. Then, the accumulated mass of precipitated antibody (*m*) at any given time point (*t*) could be modeled with for both steps separately, as follows:

(1) $$m_{\text{RETENTATE VESSEL}}(t)=\frac{dm_{\text{FEED}}}{\mathrm{dt}}-\frac{dm_{\text{BLEED}}}{\mathrm{dt}}-\frac{dm_{\text{PERMEATE}}}{\mathrm{dt}}.$$

**Figure 4:**
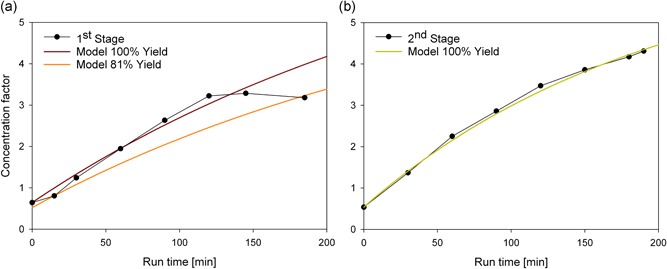
Change in the concentration factor during antibody precipitate concentration with discontinuous diafiltration. The concentration factor was the concentration of antibody relative to the concentration of antibody in the cell culture supernatant. (a) Changes in antibody precipitate concentration factors during the 1st stage. Solid lines: the expected concentration factor changes, at a yield of 100% (red) or 81% (orange). (b) Changes in antibody precipitate concentration factors during the 2nd stage, when the antibody precipitate obtained from the 1st stage was further concentrated. The first stage product was washed at a 1:10 dilution in wash buffer. Green line: the expected concentration factor change, at a yield of 100% [Color figure can be viewed at wileyonlinelibrary.com]

The antibody precipitate feed (*m*_FEED_) into the system was constant over time. We assumed that the antibody was completely precipitated; hence, the loss of antibody through the permeate (*m*_PERMEATE_) was zero. This assumption was also confirmed later, during the integration of both steps into a single process. Steady‐state occurred when the mass flow of the feed was equal to the mass flow of the bleed, and *m*_RETENTATE VESSEL_ remained constant. Knowing the initial antibody concentration, we solved the equation numerically and matched the data with experimentally determined antibody concentrations from the first stage (Figure 4a). The concentration was normalized to the cell culture supernatant concentration; thus, the curve started below 1, because the cell culture supernatant had to be diluted with the PEG6000 stock solution for precipitation. Therefore, the reported concentration factor represented the concentration relative to the culture supernatant. When we assumed 100% yield for the numerical model, the results fit very well to the measured antibody concentrations, until a run‐time of 120 min. After that, we lost antibody, and the final yield was 81%, most likely due to high antibody concentrations and formation of aggregates that could not resolubilize. The experiments to test the feasibility of sequential TFF were performed with nonoptimized buffer conditions (13.2% [w/w] PEG6000, pH 7.5). As 81% yield in antibody production is not acceptable in an antibody capture step, the integrated process was performed with optimized buffer conditions and zinc addition.

Figure 4b shows the antibody concentration over time during the second stage, where the antibody precipitate was washed with discontinuous diafiltration. As in the first stage (Figure 4a), we numerically solved the mass balance model (Equation (1)). The model fit the measured antibody concentrations very well at a theoretical yield of 100% (Figure 4b). No antibody was lost in this wash step and product loss due to high concentrations of antibody did not appear to be an issue in this stage. The higher purity during the second step compared to the first step indicated that contaminants, like HCP, DNA, and media components, were less likely to interfere with the precipitate. Once the majority of these contaminants is removed, high antibody concentrations can be achieved without losing antibody. We achieved an overall yield of 81% in both steps combined, with this sequential antibody precipitate concentration and wash procedure. We increased this yield by integrating the two units.

### 3.3 Variation of residence time in continuous precipitation

**Figure 5:**
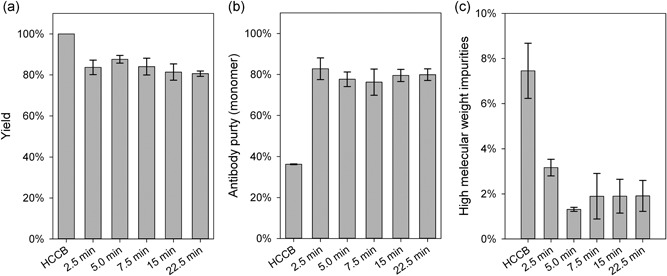
Influence of the residence time during continuous precipitation in a tubular reactor. (a) Antibody yield; (b) purity of the antibody monomer; and (c) high molecular weight impurities were measured after different residence times (2.5–22.5 min). The host cell culture broth (HCCB) served as the reference. The whiskers indicate standard deviations, which included variations in the measurements taken at different time points over the experiment period (20–160 min) and variations between experimental repetitions

A tubular reactor is necessary for a transition from batch precipitation to continuous precipitation. We have used tubular reactors in previous studies (Burgstaller et al., 2018; Hammerschmidt et al., 2016) for precipitation, but we did not quantify the influence of the tubular reactor length. Here, we assessed the impact of the reactor length (corresponding to the residence time in the reactor) during continuous antibody precipitation in the tubular reactor. We measured the impact of residence time on HMWI content, antibody yield, and antibody purity (Figure 5). The residence time in our setup was defined by the tube length and diameter and the flow rate of material through the tube. The diameter of the tube and the flow rate were constant (8 ml min^−1^); we changed the reactor length to determine whether longer or shorter reactors might lead to different process performances. Thus, we tested different residence times, ranging between 2.5 and 22.5 min, by varying the tubular length from 1.2 to 10.8 m. Figure 5 shows the effects of the various tested residence times, in terms of yield (Figure 5a), purity of the antibody monomer (Figure 5b), and HMWI content (Figure 5c). All three parameters showed stable performance, independent of the residence time. We achieved an 82.4 ± 3.5% precipitation yield and an antibody monomer purity of 80.0 ± 5.0% with nonoptimized buffer conditions in this experiment (using 13.2% [w/w] PEG6000 and pH 7.5). As 80% yield is typically not acceptable for antibody purification processes, all further experiments were carried out with the optimized buffer conditions including ZnCl_2_. Interestingly, the HMWI content was reduced significantly by the precipitation and resolubilization steps (from about 7% to about 2%), for all reactor lengths, with slight variations. We speculated that this efficiency occurred because the HMWIs precipitated easily but were difficult to resolubilize; thus, these particles remained precipitated upon resolubilization, and thus, they were lost. Because the reactor length did not seem to have a significant impact on the performance of the precipitation, we decided to use a residence time of 22.5 min, because this closely matched the incubation time of 20 min we used in all batch experiments in the present and previous studies.

### 3.4 Continuous diafiltration

Before starting the continuous operation, we tested the influence of the pore size of the hollow fiber membrane. We did not observe different results with 0.1 and 0.45‐µm pores; therefore, we selected a 0.2‐µm pore size (see Supporting Information Figure 1).

**Figure 6:**
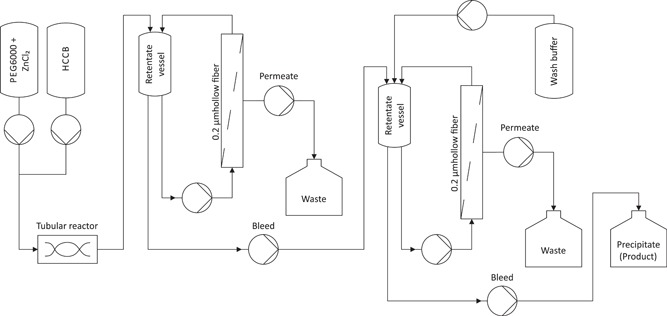
Flow diagram of continuous antibody precipitation in a tubular reactor setup. With tangential flow filtration (0.2 µm hollow fiber), the precipitate is separated (first bleed) and then washed (second bleed) in a continuous process. HCCB: host cell culture broth; PEG: polyethylene glycol

Next, we integrated all the components that we had optimized or determined into one continuous precipitation step with a TFF system (Figure 6). The optimal precipitation conditions determined in section 3.1 were used in the in‐line feed into the tubular reactor and in the feed to the retentate vessel of the first unit. A constant bleed was pumped directly into the retentate vessel of the second unit, without any holding tanks between the units, and both units were run as described in section 3.2. The bleed from the second unit was collected, and this was the concentrated purified precipitated antibody.

First, we combined the precipitant stream (PEG6000 with ZnCl_2_) with the cell culture stream in a specific ratio by adjusting the flow rates of the peristaltic pumps to achieve final precipitation conditions of 7% PEG6000 and 2 mM ZnCl_2_. The precipitation was conducted in a self‐assembled tubular reactor, with helical static mixers. These static mixers ensured a narrow residence time distribution, which prevented precipitate from settling. The length and diameter of the tubular reactor and the flow rate through the tubular reactor defined the precipitate residence time. In our case, the residence time was 22.5 min, as described above (section 3.3). After the tubular reactor, the precipitate was pumped into the first TFF unit. In a first stage, the precipitate was concentrated with tangential microfiltration (pore size 0.2 µm; membrane area 420 cm²). The precipitate stream over the membrane was 571 LMH. We selected a concentration factor of 10 (ratio of the feed flow to the bleed flow), because the concentration factor selected for previous experiments (i.e., 12.5) resulted in product loss at high precipitate concentrations. In the first 60 min of the process, the bleed into the second unit was closed off to accelerate equilibration to a steady state. After 60 min, the bleed was continuously fed into the retentate tank of stage two, and a wash buffer containing 7% PEG600 and 2 mM ZnCl_2_ was continuously added to achieve a 1:10 dilution of the bleed from the first unit.

In the second unit, again, we selected feed and bleed flow rates to achieve a concentration factor of 10, and the retentate tank level was maintained at a constant volume. Again, in this unit, we closed off the bleed to allow equilibration for a startup phase of 60 min. After 60 min (120 min after process start), the bleed out of stage‐two was started. The membrane of the second unit (pore size 0.2 µm; membrane area 110 cm²) was smaller than that of the first unit, because, in the sequential simulation process (section 3.2), we observed reduced fouling in the second stage. The precipitate stream over this second membrane was 2,182 LMH. At 180 min after the process started, we continuously harvested the washed precipitate, which was bled at a low flow rate out of the stage‐two unit.

**Figure 7:**
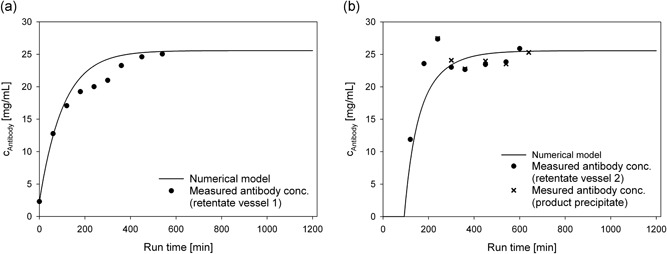
Continuous antibody precipitation with two‐stage tangential flow filtration. Plots compare the measured antibody concentrations (symbols) and the numerically solved mass balance model data (solid lines) during (a) the first stage, and (b) the second stage; filled circles: measured antibody in retentate vessel; crosses: the harvested antibody precipitate

The changes in antibody concentration during stage one run‐time are depicted in Figure 7a. The antibody concentration continuously increased up to 25.0 mg ml^−1^ during a 540‐min run‐time. In comparison, the maximum antibody concentration predicted with the numerically solved mass balance model, assuming a yield of 100%, was 25.6 mg ml^−1^. Thus, the numerically solved model fit the measured antibody concentration very well. Both the model and the measured data showed that steady state was reached after around 400 min, when the antibody concentration leveled off. The concentration factor was 8.1 compared with the initial antibody concentration in the cell culture broth before stage one. The concentration changes in the antibody and the harvested antibody during stage two are depicted in Figure 7b. Stage two started when the precipitate bleed in unit one was started after 120 min. The flow rate of the added wash buffer was the same as the flow rate of permeate removal from this unit, to maintain a constant feed inflow and product outflow in the second stage. Therefore, no additional precipitate concentration was achieved in this second stage. Indeed, this stage was dedicated as a washing step to remove residual soluble HCP and other contaminants. The continuous harvest of antibody precipitate was initiated after 180 min. After an initial spike of antibody concentration to 27.4 mg ml^−1^, the antibody concentration leveled off to roughly 25 mg ml^−1^, as expected and predicted by the model. The antibody concentration in the harvest was, as expected, analogous to the antibody concentration in the retentate tank of stage two. The model predicted that the steady state would be achieved after 400 min of run‐time. This prediction matched the experimental data. Because we started stage two with an inflow of precipitate that was more highly concentrated than the inflow of stage one, the startup phase for stage two (Figure 7b) was much shorter than that of stage one (Figure 7a). Thus, the two‐stage tangential flow microfiltration setup produced an overall concentration factor of 8.2 for the continuous harvest or antibody precipitate. The monomer yield is defined as the recovered amount of antibody over the amount of antibody, which has been fed into the system. The monomer yield of the process was 95%, after recovering precipitate from the instruments. We lost 2% of antibody monomer in the permeate of stage one, which reduced our yield; however, we did not lose a significant amount of antibody in the permeate of stage two (below 1%). We harvested 81% of the antibody when we shut it down after 600 min. After flushing the system with buffer, we recovered an additional 5% of the antibody that had remained in the tubular reactor of the continuous precipitation; another 6% that had remained in the membrane and tubes of stage one; and another 3% that had remained in the membrane and tubes of stage two. Overall, the antibody monomer recovery of the entire process was 97%. A longer process duration would most likely produce a higher yield and a more favorable relationship between the precipitate harvested at the outlet and the precipitate that was harvested by flushing the system.

**Figure 8:**
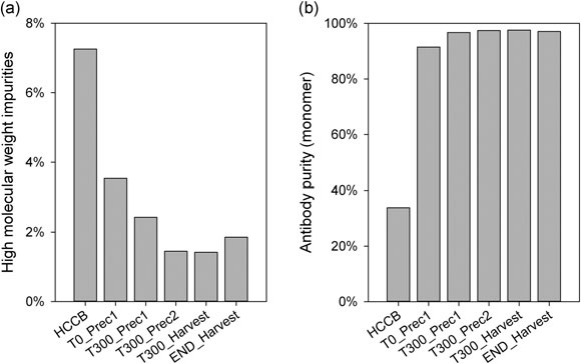
Removal of high molecular weight impurities and antibody purity measurements. (a) High molecular weight impurities in the host cell culture broth (HCCB), reduced HMWI in the resolubilized pellet in stage one (T0_Prec1) and again at 300 min (T300_Prec1). In stage two, reduced HMWI after 300 min (T300_Prec2). T300_Harvest: the precipitate harvested after 300 min; END_Harvest: pool of the precipitate harvested at the end of the process. (b) Antibody purity of the same fractions shown in (a). HMWI: high molecular weight impurities

To determine product quality and purity, we measured monomer purity and the HMWI removal with size exclusion chromatography. Unfortunately, we could not measure samples that contained ZnCl_2_ with our standard size exclusion chromatography analytics, because we observed atypical tailing. Moreover, the stationary phase of the column seemed to be affected by running ZnCl_2_‐treated samples. Even long elution steps or regeneration steps could not reverse this effect. We assumed that the stationary phase of the size exclusion chromatography column (5‐μm silica particles with diol phase groups, 250 Å pores) was not fully inert to Zn ions. Thus, the Zn ions could have mediated weak ionic interactions with proteins, and thereby constrained the elution. Although this effect might be of additional scientific interest, we did not investigate it further in the present study. To avoid this tailing, we had to wash the resolubilized samples several times to remove ZnCl_2_ before size exclusion chromatography analysis. Figure 8a shows the HMWI of various samples from the continuous run. The HMWI content was reduced from 7.3% in the initial host cell culture broth (HCCB) to 3.5% after resolubilization, at the beginning of precipitation. After 300 min of run‐time, the HMWI content was further reduced to 2.4%. After 300 min of precipitate wash in stage two, the HMWI content was reduced to 1.4%. This resulted in a fivefold reduction of HMWI compared with the initial HMWI content of the HCCB. We speculate that we might not have actually removed HMWI during the precipitation step. Instead, these impurities might have formed aggregates that were carried through the system, and they were unable to resolubilize at the end. Therefore, the HMWI were actually removed when precipitate was discarded from the resolubilized antibody. HMWI can cause severe immunological issues. Subsequent polishing steps such as (flow‐through) ion exchange chromatography, as usual in an antibody downstream process, will further reduce remaining HMWI.

The antibody monomer purity (Figure 8b) increased from an initial 34 to 91%, at the beginning of the precipitation; then, it increased again to 97% after 300 min of run‐time. This purity is comparable with that achieved in elutes from protein A chromatography; moreover, in addition, we reduced the HMWI. We expect also that the specific purification performance in regard to DNA, HCP, as well as nature of HCP, can match the performance of protein A chromatography, as was already shown in other publications (Hammerschmidt et al., 2016; Sommer et al., 2015). To achieve continuous protein A affinity chromatography, the process must be cycled or performed with counter‐current loading. However, that method interrupts the continuous mass flow, and in turn, it significantly increases the residence time distribution of the entire process. In contrast, our process provided continuous, uninterrupted mass flow, and a fully continuous process could be maintained. Furthermore, the outflow of the resolubilization reactor could be fed directly into a continuous virus inactivation solution, (Klutz, Lobedann, Bramsiepe, & Schembecker, 2016) and subsequent flow‐through chromatography (Ichihara, Ito, Kurisu, Galipeau, & Gillespie, 2018) would not interrupt the mass flow.

The process described here had several advantages over other methods. First, it incorporates a simple, robust setup of readily available instrumentation, because TFF is a standard unit operation currently used in the pharmaceutical industry for buffer exchanges, perfusion systems, and other operations (Rucker‐Pezzini et al., 2018). Second, precipitation is, by nature, a concentration‐independent process; the addition of precipitant only depends on the volume that must be processed, not on the specific antibody concentration in the feed stream. Thus, a very simple control loop is required to maintain steady‐state precipitation, and it is not necessary to perform complex, expensive on‐line measurements of antibodies in a complex feedstock. This feature greatly simplifies the purification process, particularly in the context of perfusion cultures with changing antibody concentrations. Third, our TFF‐based precipitation method provided a continuous steady outflow of product. This steady stream could be directly connected to the subsequent unit operations without intervening surge tanks.

## 4 CONCLUSION

In this study, we developed a truly continuous antibody capture process. The antibody was continuously precipitated from clarified culture supernatant, and the antibody precipitate was continuously concentrated and washed with yield and purity similar to those achieved with protein A affinity chromatography. This process was robust because fluctuations in the feed stream were readily handled. There was no need for on‐line protein concentration monitoring. The setup could be readily realized as a disposable unit, because the necessary equipment, such as tubing, fittings, static mixers, and hollow fiber modules are commercially available. This process is truly continuous compared with other, quasi/semicontinuous chromatography processes, which require a cyclic operation. Our process did not require surge tanks. This process could be used to integrate capture, virus inactivation, and even first intermediate purifications.

## CONFLICTS OF INTEREST

The authors declare that there are no conflicts of interest.

## Supporting information

Supporting information
