# Supplementary material for: Continuous integrated antibody precipitation with two‐stage tangential flow microfiltration enables constant mass flow
Source: Biotechnol Bioeng. 2019 Jan 23;116(5):1053–65. doi: 10.1002/bit.26922 (PMC6667901; doi:10.1002/bit.26922)
Supplement: Supplementary file 1 — Supporting information [file BIT-116-1053-s001.docx]

Supporting information

## Variation of membrane pore size

In preliminary experiments, we found that the capillary within the hollow fiber became blocked, due to the high viscosity of the concentrated precipitate. To address this potentially limiting factor, we tested two different hollow fiber microfiltration membranes with pore sizes of 0.1 µm and 0.45 µm to evaluate the difference in filtration properties. During our TFF microfiltration setup, the permeate flow was constrained by a pump, and not governed by the TMP; therefore, the TMP did not provide conclusive information. Instead, we monitored the feed pressure of the membrane and the antibody concentration at different time points throughout the run-times of two different batch precipitate concentration steps, one with a 0.1‑µm membrane, and one with a 0.45-µm membrane (Fig. 9). We observed similar feed pressures for both membranes. Nevertheless, a slightly earlier increase in feed pressure was observed with the 0.1‑µm compared to the 0.45‑µm membrane, but the difference was very minor. The antibody concentrations measured at all time points were the same with both membranes, except the samples measured at 60 min. At that time point, the antibody concentration was 30.3 mg mL^-1^ (concentration factor of 8.8) with the 0.1-µm membrane, compared to 21.3 mg mL^-1^ (concentration factor of 6.2) with the 0.45-µm membrane. A potential explanation for this difference might be that, at high concentrations, the precipitate might have been quite viscous, which reduced the accuracy of the flow rates controlled by the peristaltic pump and the sampling was more error-prone. To rule out the possibility that the antibody was lost in the permeate, we also measured the concentration in the permeate, but there was no difference between membranes (1.4% loss through both membranes). We selected the 0.2-µm pore size membrane because, according to additional data on process parameters, it provided the best tradeoff between concentration and viscosity; thus, it could achieve the highest possible concentration (Supporting Figure 1) and avoid constricting the flux through the membrane. In addition, previous work showed that 0.2-µm pore size filter devices provided good performance for batch operated precipitation ([Hammerschmidt, Hobiger, & Jungbauer, 2016](#_ENREF_1)).


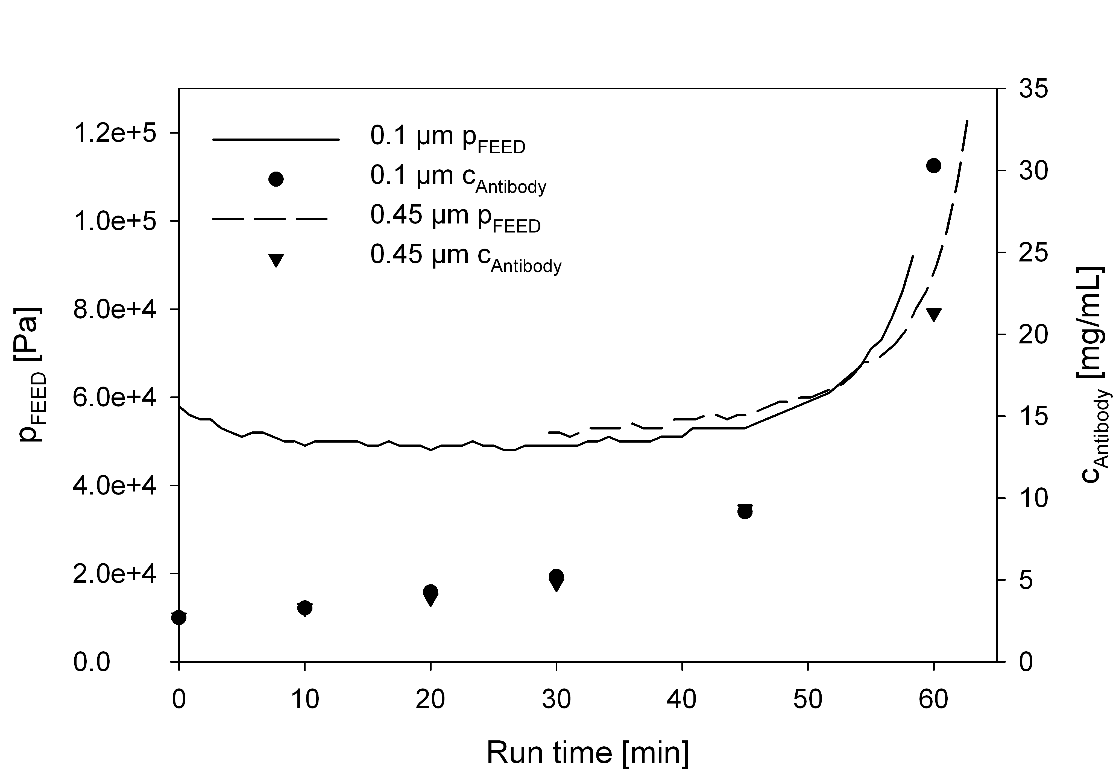


Supporting Figure 1. Antibody precipitate concentrations with different filters in tangential flow filtration. The membrane feed pressures (lines) and antibody concentrations (symbols) were measured over time. The hollow fiber membranes had pore sizes of 0.1 µm (solid line, filled circles) and 0.45 µm (dashed line, filled triangles).

# References

Hammerschmidt, N., Hobiger, S., & Jungbauer, A. (2016). Continuous polyethylene glycol precipitation of recombinant antibodies: Sequential precipitation and resolubilization. [Article in Press]. Process Biochemistry, 51(2), 325-332. doi: 10.1016/j.procbio.2015.11.032
